# The Response of HeLa Cells to Fluorescent NanoDiamond Uptake

**DOI:** 10.3390/s18020355

**Published:** 2018-01-26

**Authors:** Simon R. Hemelaar, Babujhi Saspaanithy, Severin R. M. L’Hommelet, Felipe P. Perona Martinez, Kiran J. van der Laan, Romana Schirhagl

**Affiliations:** Department of Biomedical Engineering, University of Groningen, 9713 AV Groningen, The Netherlands; simonhemelaar@gmail.com (S.R.H.); babujhi.s@gmail.com (B.S.); severin.l.hommelet@etu.univ-poitiers.fr (S.R.M.L.); felipeperona@gmail.com (F.P.P.-M.); kiranvanderlaan@gmail.com (K.J.v.d.L.)

**Keywords:** fluorescent nanodiamonds, reactive oxygen species, cellular uptake, biocompatibility

## Abstract

Fluorescent nanodiamonds are promising probes for nanoscale magnetic resonance measurements. Their physical properties predict them to have particularly useful applications in intracellular analysis. Before using them in intracellular experiments however, it should be clear whether diamond particles influence cell biology. While cytotoxicity has already been ruled out in previous studies, we consider the non-fatal influence of fluorescent nanodiamonds on the formation of reactive oxygen species (an important stress indicator and potential target for intracellular sensing) for the first time. We investigated the influence of different sizes, shapes and concentrations of nanodiamonds on the genetic and protein level involved in oxidative stress-related pathways of the HeLa cell, an important model cell line in research. The changes in viability of the cells and the difference in intracellular levels of free radicals, after diamond uptake, are surprisingly small. At lower diamond concentrations, the cellular metabolism cannot be distinguished from that of untreated cells. This research supports the claims of non-toxicity and includes less obvious non-fatal responses. Finally, we give a handhold concerning the diamond concentration and size to use for non-toxic, intracellular measurements in favour of (cancer) research in HeLa cells.

## 1. Introduction

While nanodiamonds have been widely used as abrasives for decades, new applications have been discovered recently. In Fluorescent Nanodiamonds (FNDs), a Nitrogen Vacancy (NV) centre is created which can be utilized as stable single photon emitter [1] or spin qubit in quantum information [2]. The spin properties of diamond defects are utilized in nanoscale sensors. These can detect magnetic resonances [3,4], pressure [5], temperature [6] or electric fields [7] with unprecedented spatial resolution [8]. In addition, nanodiamonds are under consideration for drug delivery [9,10] and photostable fluorescence labelling [11,12]. As a result, there is a broad interest in studying the (bio)compatibility of nanodiamonds. Their internalization into different cell types [13,14,15,16,17,18] and lack of cytotoxicity [19,20,21] have been confirmed in several studies in different cell models. We confirm this finding and see virtually no effect on cell viability. FND toxicity in vivo has been found to be very low or negligible repeatedly in different organisms [22,23]. However, Marcon et al. found a decrease in the rate of survival of frog embryos after administration of relatively high concentrations of 2 mg/mL of 4 nm detonation nanodiamonds. In most rodent studies (mouse or rats) no clinical symptoms can be found of either small or large nanodiamonds and the diamonds tend to accumulate in lung and liver tissues [24,25,26]. In Cynomoglus monkeys, some abnormalities were seen in histological evaluations of the heart and liver, using detonation nanodiamonds at a high concentration of 25 mg/kg bodyweight [27]. Some more toxic effects have been found for detonation nanodiamonds [28,29], while other studies report no cytotoxic effects depending on the surface treatment [30,31]. FNDs and detonation nanodiamonds have very different properties due to size, surface to volume ratio and surface termination. Detonation nanodiamonds are not the topic of this study. Non-toxic influences are very important since these might still alter the cell biology. Such influences have only been taken into account in very few studies. Thomas et al. for instance studied the expression of genes related to inflammatory responses and observe down regulation of these genes (at a relatively high diamond concentration of 50 µg/mL) [32]. Moore et al. investigated genes involved in proliferation, inflammation and apoptosis and did not see any differences compared to the control sample [33]. Huang et al. found no indication of cell death but did see morphological changes in neurons after exposure to FNDs [34]. In malignant cell growth, free radical levels are an important determining factor [35]. Mohan et al. investigated overall reactive oxygen species (ROS) levels in *Caenorhabditis elegans* [36]. They did not find altered ROS levels, nor did they detect any genotoxic effects.

The goal of this study is to assess in detail if FNDs are suitable for intracellular sensing and what non-fatal impact the presence of diamond particles has on a cell. We chose to study HeLa cells since they are a very common cell model for various types of research. When the term biocompatible is used in this paper, this refers specifically to compatibility for HeLa cells. We provide a detailed analysis of non-fatal influences of diamond on the reactive oxygen species formation in cells for the first time. This is particularly relevant, for two reasons. First, they are an attractive analyte for sensing applications and second they indicate oxidative stress. 

## 2. Materials and Methods 

### 2.1. Cell Culturing

HeLa cells were cultured in Dulbecco’s Modified Eagle Medium with 4500 mg/L glucose (DMEM-HG), supplemented with 10% Foetal Bovine Serum (FBS), 1% Penicillin/ streptomycin and 1% Glutamax (Gibco, ThermoFisher Scientific, Etten-Leur, The Netherlands) at 37 °C, 5% CO_2_. HeLa cells are a favourable model in (cancer) research, as these are an extensively studied cancer cell line. Cells were seeded in gamma irradiated 35 mm glass bottom collagen coated dishes (MatTek corporation, Ashland, MA, USA) until clusters of at least 10 cells grew for confocal microscopy. For mRNA and protein analysis, cells were grown in 6-wells plates (Greiner Bio-One, Frickenhausen, Germany). For the MTT (3-(4,5-dimethylthiazol-2-yl)-2,5-diphenyltetrazoliumbromid) viability assay and total free radical analysis, cells were grown in Greiner 96-wells flat bottom plate.

### 2.2. Diamond Uptake

From Petr Cígler, IOCB Prague, we received etched diamond particles [16] (with rounded edges). The other diamonds were obtained from Adámas Nano (Raleigh, NC, USA), see also Table 1. Nanodiamonds were first suspended in 100 µL 100% FBS-HI (Heat-Inactivated Foetal Bovine Serum) to prevent aggregation, as shown previously [13]. Next, 900 µL DMEM-HG was added and the diamond suspensions were incubated with precultured cells for 5 h at 37 °C, 5% CO_2_. We used concentrations, which ensured that at least every cell had multiple intracellular diamonds. When performing Electron Spin Resonance (ESR) measurements, it is preferable to have one or a handful of nanodiamonds per cell, as too much diamonds will make it more difficult to obtain the spectra of a single particle/NV centre. Therefore, we chose to use 10 µg/mL of nanodiamonds as an upper limit, as this results in already more diamonds per cell than useful for quantum measurements. One sample was taken into account in which the particles were added directly to DMEM-HG supplemented with FBS and thus aggregation was not prevented. As a positive control for cellular damage, cells were incubated with 1 mM, 200 µM or 40 µM H_2_O_2_ (hydrogen peroxide) for 2 h. Afterwards, the diamond or H_2_O_2_ containing medium was removed and the cells were used for microscopic visualization or different analysis methods, see below. To investigate the long-term influence of the diamonds as well as possible recovery from an impact, we also tested the cells after incubating them for 24 more hours (T = 24) in supplemented DMEM-HG medium without diamonds or H_2_O_2_ before further analysis.

### 2.3. Microscopic Analysis

We performed a microscopic analysis to identify and quantify ingested diamond particles. Cells were fixed in 3.7% PFA (Paraformaldehyde) and subsequently blocked in 5% PBSA (bovine serum albumin in phosphate buffered Saline). After blocking, we used 2 µg/mL phalloidin-FITC (Sigma-Aldrich, Zwijndrecht, The Netherlands) to label f-actin and 4 µg/mL DAPI to label the nucleus (Sigma-Aldrich, Zwijndrecht, The Netherlands) in 1% PBSA. The samples were imaged using a LSM780 confocal microscope (Zeiss, Sliedrecht, The Netherlands) using a 405, 488 and 561 nm laser. Images were analysed using FIJI 2.0.0 software (https://fiji.sc). A visual inspection of the morphological changes was performed to estimate the effect of the diamond uptake on the cells cytoskeletal condition. Next, a specific, custom-made FND quantification plugin was used to approximate the amount of internalized FNDs. The analysis was divided into three phases: Cell Selection, Masking and Particle Analysis. During the first phase, the images were visually inspected and random cells were selected for the analysis. Cells with diamond aggregates associated with the cell membrane were rejected to prevent false positive results. The images were composed of several slices (Z-stacks) and the cellular region was defined in all the three dimensions. In the horizontal plane, the selection considered an area containing only the cell of interest. In the height, the first and last slices containing the cell were identified. As a result, the first phase defines a volume that holds only the cell of interest. In the Masking phase, that volume is moulded in order to resemble the shape of the cell. The phalloidin-FITC signal is converted to binary using the Isodata algorithm to calculate the threshold [37] and the cell’s perimeter is detected in every slice. To find the inner volume of the cell, the program shrinks the cell’s region in order to exclude the cell membrane from the analysis. The final step uses a special function of Fiji, which analyses the particles found in a selected region. Applying this function to the masked image, it is possible to directly obtain the number of objects (connected positive pixels) in the specified region. A threshold is used to separate the background light from the signal emitted by the FNDs. Every pixel with intensity less than the threshold is assumed as background and set as black, while every pixel with an intensity greater than or equal to the threshold is assumed as part of a particle. To find an adequate value for this parameter, the image was visually inspected and different values were probed. Finally, we chose the lowest possible value, which gives zero for a negative control image. In the end, this method gives two important values: the number of objects, which reflects the amount of adjacent FND positive pixels, where a single diamond or multiple diamonds can be counted as 1 and the number of particles, which reflects the actual number of particles by calculation from the intensity and size of the objects.

### 2.4. Cellular Viability

To test the viability after incubation with FNDs, HeLa cells were washed once with PBS (phosphate buffered saline). Next, 0.05% MTT (3-(4,5-dimethylthiazol-2-yl)-2,5-diphenyltetrazoliumbromid, Sigma Aldrich, Zwijndrecht, The Netherlands) and serum-free medium were added to the cells. After two hours of incubation at 37 °C, 5% CO_2_, the cells were washed with PBS. Subsequently, the cells were dissolved using 2-propanol and the absorption of the purple solution was measured using a FLUOstar Omega Microplate Reader (BMG Labtech, De Meern, The Netherlands) at 560 nm. After correction and comparison to the background, this gives a ratio of the viability of the cell, where 0.8–1.2 is considered to be the normal range [38]. Results of MTT were validated using light microscopy.

### 2.5. Total ROS Activity 

2′,7′-dichlorodihydrofluorescein diacetate (DCFDA) can be used as an indirect measure for the total ROS production inside a cell. After entering the cell, DCFDA is deacetylated and later oxidized by ROS to 2′,7′-dichlorodihydrofluorescein (DCF) which is highly fluorescent. First, 20 µM DCFDA in phenol red-free DMEM medium is added to the cells and incubated for 45 min. Then the cells were incubated with FNDs. Incubation with 50 µM tert-butyl hydroperoxide (TBHP) instead of FNDs is used as a positive control. HeLa cells without a stimulant were used as a negative control. In case of T = 24 h measurements, TBHP was added 4 h and DCFDA 45 min prior to the end of the incubation time. Fluorescence was measured directly after incubation using a FLUOstar Omega Microplate Reader, excitation 485 nm and emission 520 nm. All samples were related to the negative control after subtraction of the background (medium without cells) and shown as a fold increase. A separate negative control was made for cells after 24 h. All samples were performed in triplicate. While the DCFDA analysis gives a good measure for the overall ROS production, quantitative polymerase chain reaction (qPCR) and western blotting measures the cells response to these stress factors.

### 2.6. RNA Isolation and Real-Time PCR 

qPCR was used to evaluate intracellular mRNA transcription levels of Catalase (CAT), Glutathion Reductase (GSR), SuperOxide Dismutase 1 (SOD1) and Caspase-3 (CASP). These are common enzymes, which are expressed as a response to oxidative stress. More precisely, superoxide dismutase 1 is involved in the conversion of singlet oxygen radicals to hydrogen peroxide. Catalase and glutathion reductase are both involved in metabolizing H_2_O_2_ into non-toxic compounds like water or oxygen. Caspase-3 is a marker for apoptosis. Total RNA was isolated from cell cultures using the InVisorb Spin Cell RNA Mini Kit (InVitek, GmbH, Berlin, Germany), following the manufacturer’s instructions. The quantity and purity of RNA were determined using a spectrophotometer (NanoDrop, Wilmington, DE, USA). RNA was reverse-transcribed using the I-script reverse transcription kit (Bio-Rad Inc., Hercules, CA, USA) while following the manufacturer’s instructions. Real-time polymerase chain reaction (PCR) amplification of cDNA was performed using the Sybr green mix from Abgene (Westburg BV Leusden, The Netherlands) and specific oligonucleotide primers listed in Table 2. The real-time PCR parameters were as follows: 95 °C for 15 min, then 40 cycles at 95 °C for 15 s, 57 °C–60 °C for 15 s and 72 °C for 15 s. Data were analysed using the 2^−ΔΔCT^ method of Livak and Schmittgen [39], using the housekeeping gene 18S (a gene which encodes for ribosomal RNA and is present in the same amount in every cell) to calculate the ΔCT and using the control at each measurement to calculate the ΔΔCT. This value gives a measure to quantify the genetic response in relation to the basic metabolism in the cell. All samples were performed in triplicate and measured in 3 independent qPCR runs.

### 2.7. Western Blot

PCR shows the production of mRNA in response to an impact. This is a first, fast response of a cell and thus reflects the ‘intentions’ of the cell. However, the cell has also epigenetic self-correction mechanisms. As a result, not all mRNA is actually translated to proteins. The protein level, which we evaluated via western blot, gives a better measure of the current situation in the cell. Western blot analysis was used to evaluate intracellular protein levels of catalase, glutathion reductase, superoxide dismutase 1 and caspase-3. For this, HeLa cells were harvested in denaturation buffer (10 mM Tris-HCl pH 7, containing 1 mM EDTA, 2.5% SDS, 2% 2-mercaptoethanol and 10% glycerol) and proteins were analysed by SDS-PAGE according to the method of Laemmli using a 11% running gel as previously described [40]. After separation, the gel was blotted to nitrocellulose and blocked for 1 h with 3% BSA, 0.1% Tween in TBS (tris buffered saline). After incubation overnight at 4 °C with the primary antibody, different secondary antibodies (listed in Supplementary Table S1) were added and allowed to incubate for 1 h. Then, the blot was incubated with alkaline phosphatase(AP)-conjugated tertiary antibody diluted 1:1000 for another hour. After washing, the blot was developed with nitro blue tetrazolium and 5-bromo-4-chloro-3-indolyl phosphate in AP buffer. All incubation and washing steps were performed at room temperature, unless stated otherwise. The primary antibodies rabbit anti-caspase 3, anti-superoxide dismutase 1, anti-catalase and anti-gluthation reductase (Supplementary Table S1) were further incubated with mouse anti rabbit IgG (Jackson ImmunoResearch Laboratories Inc., West Grove, PA, USA) first and then with goat anti-mouse AP (Bio-Rad Inc., Hercules, CA, USA). Mouse anti-tubulin and anti- glyceraldehyde-3-phosphate dehydrogenase, two housekeeping proteins, used as a loading control (Supplementary Table S1), were incubated with goat anti-mouse IgG (Jackson ImmunoResearch Laboratories Inc., West Grove, PA, USA) and then with rabbit anti-goat AP (Bio-Rad Inc., Hercules, CA, USA). The ratio between the protein of interest and the loading standard tubulin or 18S was calculated using FIJI software. All samples were performed in triplicate and measured in 3 independent Western Blots.

## 3. Results

### 3.1. Uptake of FNDs in HeLa Cells

**Figure 1:**
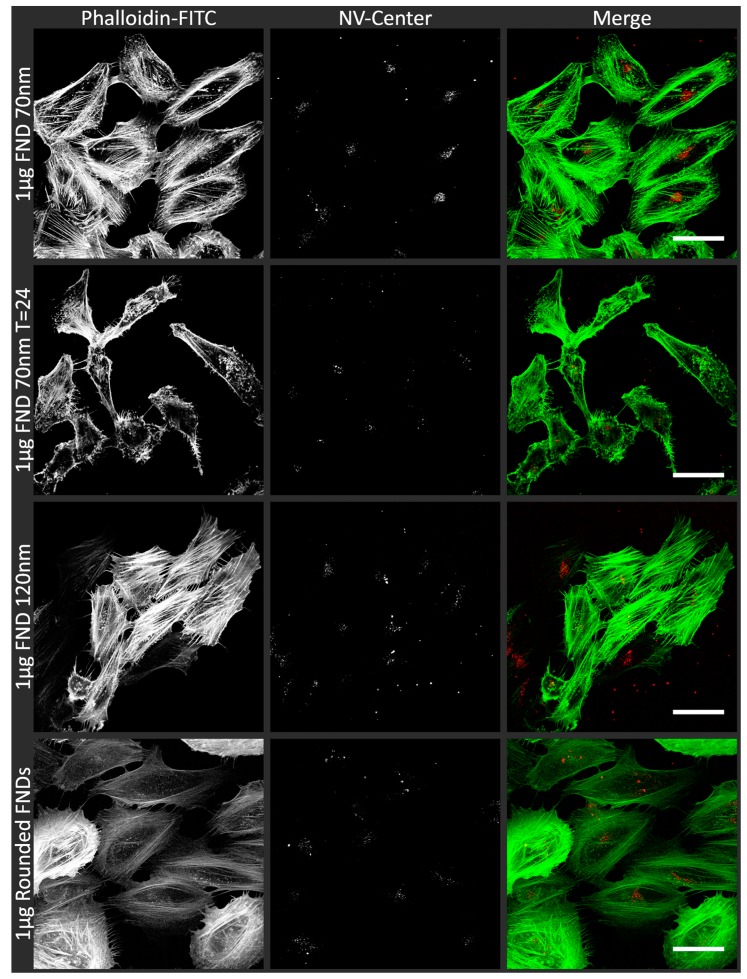
Confocal images of HeLa cells incubated with FNDs. To visualize internalized nanodiamond particles (in red), HeLa cells were fixed with 3.7% PFA and subsequently stained with Phalloidin-FITC and imaged using a LSM780 microscope. Phalloidin-FITC stains the actin cytoskeleton of the cells (in green). For visualization purposes, both signals are shown in white and merged. As examples, cellular uptake of the standard situation (1 µg FND_70_), the uptake after 24 h, cells with 120 nm FNDs (1 µg FND_120_) and cells with rounded FNDs are shown. The morphological differences between the cells in the images are a natural variation. As each cluster grows out of one mother cell, they resemble the closest sister cells but not by definition the cells of other clusters. The images were recorded in z-stacks and a focal plane was chosen to display here, approximately 2 μm above the cover glass, as these show the largest volume of the cells. The scale bars in these single optical section images represent 50 μm.

**Figure 2:**
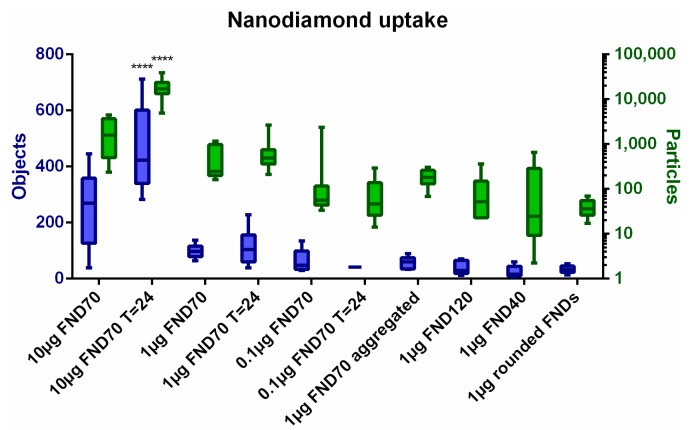
FND uptake in HeLa cells. After confocal imaging, cells are analysed using FIJI software. Our analysis counts the objects and particles inside cells, giving an arbitrary measure for the amount of diamonds taken up. Objects are adjacent FND positive pixels in cells, incorporating both single particles and aggregates or adjacent particles. Particles represent an estimation of the amount of FNDs, calculated by the intensity and size of the objects. The sample incubated with more nanodiamonds, 10 µg of 70 nm FNDs T = 24, resulted in significantly more nanodiamonds per cell (*p* < 0.001) in comparison to most other samples with the exception of 10 µg of 70 nm FNDs T = 0.

The uptake of differently shaped and sized FNDs, as well as the uptake following incubation for 24 h, was visualized using confocal microscopy. We used FIJI software with a homemade script to define objects and particles as an arbitrary measure to quantify the internalized particles. In Figure 1, examples of the confocal images are shown. We selected our ‘standard’ quantity of diamonds, 1 µg of FND_70_, immediately after uptake and 24 h after uptake. Also, FNDs of a different size (FND_120_) and shape (rounded FNDs) are shown. The shape of the particles we used in this study have already been characterised elsewhere. To confirm the rounded and prickly shape we would like to refer the reader to SEM images in references [16,41].We deliberately made a selection of incubation conditions as showing all of these overall gives the same idea. In these images sometimes multiple adjacent pixels are positive for FND. This can be explained by aggregation of particles, a limited resolution (the pixel size here is approximately 1 µm) or colocalization of the diamonds in for example endosomes. The variation of ingested objects/particles between the different incubation conditions is quantified in Figure 2, the original images from which this data was calculated can be seen in Supplementary Figure S1. As can be expected, the amount of diamonds added to the samples also results in a corresponding increase or decrease of the number of objects inside a cell. 10 µg /mL of diamonds results in a significantly higher concentration of internalized objects. The different sizes and shapes of diamonds do not result in a rigorously altered ingestion. The difference between the immediate analysis and the analysis after 24 h is not significant. An image of the cells without diamonds (data not shown) resulted in 0 particles/cell. It also has to be noted, that the time intervals for cell divisions for these cells is around 22 h [42]. During the 24 h further incubation time, some cell divisions will have taken place and hence there are more cells, which influences the results at T = 24. The diamonds seem to be distributed equally over all cells, indicating that there is no selection process for the diamonds during cell division. The amount of FNDs inside the cells does not change significantly over 24 h, indicating that there is no excretion of the particles. In Supplementary Figure S5 and Table S3 we give an approximation of the morphological changes at different time points after uptake of different concentrations of 70 nm FNDs, in comparison to a control (no diamonds).

### 3.2. Biocompatibility of Nanodiamonds

**Figure 3:**
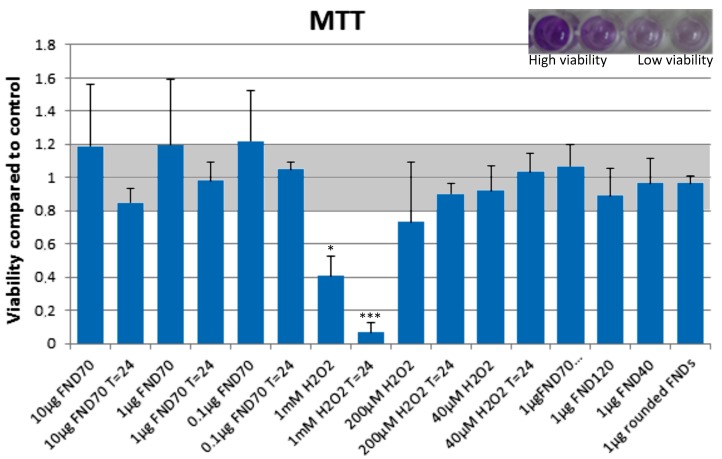
Viability of cells after FND uptake. If the viability compared to the control is between 0.8 and 1.2 it is considered to be unaffected. The samples in which 1 mM H_2_O_2_ was used differed significantly from the control (* *p* < 0.05, *** *p* < 0.001). Error bars show the standard deviation.

The viability of cells in all different conditions of nanodiamonds and H_2_O_2_ were tested using a MTT assay. MTT is converted by mitochondrial reductase enzymes to formazan, which has a purple colour. This process only happens if the cell is alive and metabolically active. The results of this analysis can be found in Figure 3. The inset in Figure 3 shows the dark purple solution resulting from viable cells, whereas the colourless solution indicates non-viability. It is important to note that the viability is generally considered equal to the control if it is between 0.8 and 1.2 times the control values. Thus, we can conclude that the viability of cells after diamond uptake is not changed in any of our experiments. Hydrogen peroxide was used as a positive control to directly be able to measure the effects of an increased concentration of oxidative products. We have also tested the production of free radicals and viability after administration of different concentrations of LPS, as this can increase cellular oxygen radical production [43]. This however did not lead to a sufficient overall free radical production to show the desired effects, see Supplementary Figure S2 (Free radical production) and Supplementary Figure S3 (Viability). Therefore, we chose a series of hydrogen peroxide concentrations to better evaluate the cellular response. The shape, size or concentration of the diamonds does not influence the viability of the cells. All samples after 24 h are also in the normal viable state, with the exception of the positive control were high concentrations of hydrogen peroxide were added. 

### 3.3. Total ROS Activity 

**Figure 4:**
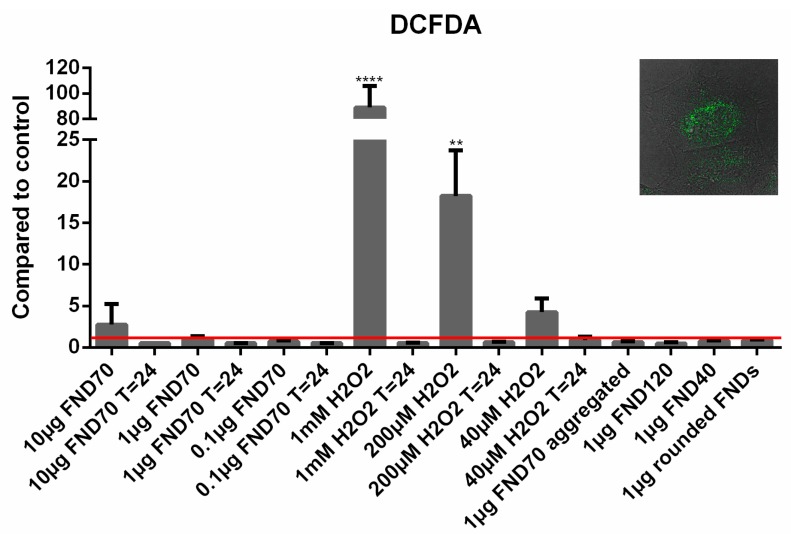
Mean free radical production. ROS production can be measured by the conversion of DCFDA to DCF. The more DCF there is, the higher the fluorescent signal a sample emits. As expected, adding a high concentration of hydrogen peroxide increases the signal drastically (up to 100-fold). All diamond samples do not alter the total free radical production inside cells. HeLa cells without a stimulant were used as a negative control to relate all values to. ** *p* < 0.01, **** *p*< 0.0001. Error bars show the standard deviation. The inset in this figure shows a cell in greyscale with the metabolized DCF in green.

To evaluate the total ROS production inside HeLa cells, we evaluated the free radical dependent conversion of DCFDA into its fluorescent metabolite DCF. The fluorescence of each sample was compared to the negative control (Figure 4). As can be expected, adding high concentrations of hydrogen peroxide to the cells increases the fluorescence drastically and significantly. After 24 h we do not see this increase; by then all the free radicals have reacted to other compounds, damaging the cells. The FNDs do not alter the total ROS production significantly, however a slight, non-significant decrease in ROS activity was found in the samples after 24 h and in the samples with 120 nm FNDs (see Supplementary Figure S4). This may suggest a free radical scavenging function of the diamonds.

### 3.4. Real-Time PCR 

**Figure 5:**
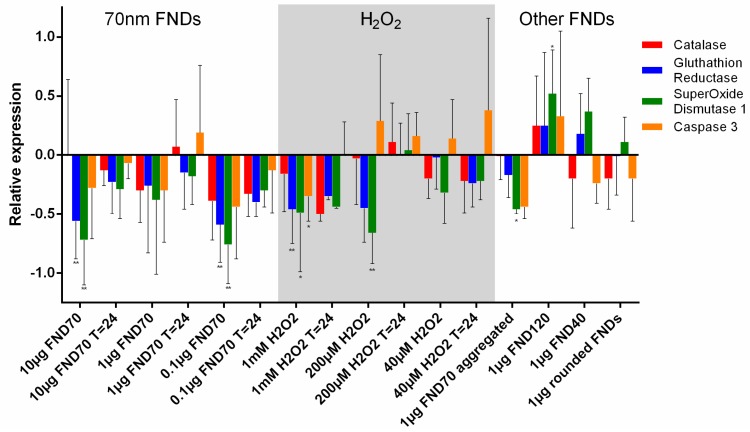
Relative expression of oxidative stress-related genes. The relative expression of four different genes as a response to the uptake of diamonds or the presence of H_2_O_2_ has been analysed using quantitative PCR. The control is set as zero, the increase or decrease of the genetic expression is showed for all samples. Glutathion reductase and superoxide dismutase differed most often significantly from the control. * *p* < 0.05, ** *p* < 0.01. Error bars show standard deviation. Values are averages out of three independent qPCR runs that were performed in triplicate.

For a closer look at the cellular response to diamond uptake, we analysed the changes in mRNA levels. mRNA indicates a cell’s first response, as the nucleus transcribes those parts of the DNA that are needed at that time. In general, a downregulation of genes involved in the scavenging of free radicals can be seen (Figure 5). For diamond particles, we see a larger down regulation for oxidative stress-related genes. When comparing aggregated to non-aggregated particles or different concentrations we do not see any significant changes. In the case of hydrogen peroxide, we see an upregulation of caspase-3 at lower concentrations (200 µM and 40 µM), which indicates an onset of apoptosis. At the highest hydrogen peroxide concentration (1 mM) we see a slight downregulation of caspase-3, which is probably due to a progressed stadium of apoptosis. As can be seen in the MTT assay, (Figure 3) many of the cells have already died at this point. 

### 3.5. Protein Transcription

**Figure 6:**
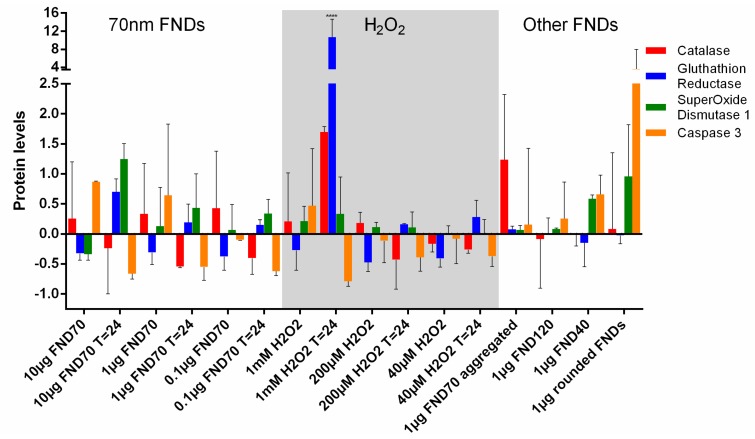
Levels of oxidative stress-related proteins. By quantitating Western Blots, protein levels as a response to the uptake of diamonds or the presence of H_2_O_2_ have been analysed. The control is set as 1, the protein levels are shown as fold increase or decrease for all samples. The protein levels after uptake of 40, 70 and 120 nanometre FNDs did not differ significantly from the control situation. Note that although in cells with rounded FNDs the caspase-3 levels are increased, this is not significant. **** *p* < 0.0001. Error bars show the standard deviations. The values are averages out of 3 independent Western Blots of samples in triplicate.

By analysing Western Blots the protein levels in cells can be measured. This reflects the current situation inside the cell, whereas the measurement of mRNA levels reflects the “intentions” of the cell. Western Blot proves a useful tool to observe if the altered mRNA levels also affect cellular protein levels. This is visualized in Figure 6 and Supplementary Table S2, the latter correlating the (non-significant) changes of both the qPCR and the Western Blot. Generally, the differences in protein levels are less pronounced than in mRNA levels. This can be due to epigenetic changes of the mRNA or a feedback mechanism on the protein. We also see drastic changes in protein expression in the sample with 1 mM H_2_O_2_. Here the surviving cells clearly have produced a high abundance of GSR and catalase to deal with the peroxide. This demonstrates that the protein response of the cells to oxidative stress requires some time to be fully effective. Catalase and glutathion reductase play a smaller role in the samples where diamond particles were internalized. In the control situation, no FND incubation or H_2_O_2_ treatment of the cells took place.

## 4. Discussion

Here we have for the first time studied the non-fatal response to nanodiamond uptake for diamonds with different sizes, shapes and concentrations. Although there are some changes to the cellular mRNA and protein levels, the overall data indicate that diamonds are biocompatible and do not negatively influence the total free radical level inside the cell. Furthermore, while we see slight changes immediately after the uptake, cells recover very well from the ingestion after 24 h. HeLa cells respond especially well to concentrations of 1 µg/mL FND_70_ and lower, 24 h after uptake. At this time point, the smallest genetic and protein differences are found. A normal viability level, as well as a conventional number of oxidative products in the cells, also argues for an ideal culturing condition. On the morphological level, the cytoskeleton of the cells seems to be mostly normal after 24 h, although some indication of an early onset of apoptosis can be seen. This however happens also under control conditions. Another explanation can be found in the microscope setup, where a different focus or different light intensity can show a slightly altered picture. Typically, 24 h is long enough for most FND related experiments but to rule out longer term viability difficulties, cells could be monitored after 48 h. Using a concentration of 1 µg/mL FND70, every cell has at least tens of internalized objects; for most magnetic resonance measurements 1 nanodiamond per cell is already sufficient. The internalized diamonds were calculated after excluding diamonds in the membrane region. It is possible that the diamonds attached to the membrane also influence the cellular behaviour. If some cells would have only a few diamond particles ingested the effect on the cell biology could be too low to measure, which is why we chose higher than necessary concentrations. Our results therefore overestimate the effects for the desired application. 

Most stress-related genes are down-regulated after incubation with nanodiamonds. This could have basically three reasons: (1) the number of radicals is decreased by scavenging, so that the cells have less compensation needed for an excessive radical production, (2) the cells are going into apoptosis or (3) a temporary increase in stress at a prior point before FND incubation is completed results in an overshoot and down-regulation of scavenging related proteins. To differentiate the cases, we can compare the levels at 24 h after uptake as well as the results from the MTT assay. The fact that the values generally decrease after 24 h indicates that the cells are recovering (as opposed to the positive control). There are no signs of decreasing metabolic activity visible in the MTT assay, which underlines the suggestion that downregulation could be due to a scavenging effect. Diamonds of 120 nm in diameter do cause a significant up-regulation of superoxide dismutase-1. The up-regulated transcription does not result in a higher protein translation however, as can be deducted from Figure 6.

In a previous study, we have extensively studied the formation and prevention of FND aggregates for applications in cell culturing [13]. Confocal images revealed that even in samples where aggregation was prevented, diamonds seemed to cluster. It has to be noted that conventional confocal microscopy is diffraction limited. Multiple diamonds can be located in the same pixel, without being aggregated. Super resolution confocal microscopy or electron microscopy can be used to more accurately analyse this aggregation. Aggregated diamonds enter HeLa cells in a similar fashion to their single counterpart. This is true for HeLa cells which more easily take up particles but will not be true for cells which are less prone to take up larger particles, such as colon carcinoma cells [44]. 

The exact localization of nanodiamonds is an interesting phenomenon. Chu et al. showed that nanodiamonds escape to the cytoplasm [16] but did not give an indication to which organelle the diamonds are ultimately localized. Lake and Bouchard very thoroughly demonstrated that they were able to target nanodiamonds toward the nuclear pore complex [45] and Chan et al. directed the diamonds towards the mitochondria [46]. During our confocal analysis, we found that diamonds seem to localize near the nucleus. Future research should point out if and to what extent and distance the nanodiamonds colocalize with cellular organelles. 

Next to advancements and opportunities for in vitro applications of nanodiamonds, like monitoring magnetic resonances and temperature, nanodiamonds have also shown potential for biomedical purposes by numerous in vivo studies (reviewed in [47]). Within the nanomedicine field, one of the applications that has attracted increasing attention is drug delivery. Using nanodiamonds in drug delivery systems aims to improve stability of drugs in physiological environment and to increase targeting efficiency and localized drug release [48,49,50]. Another purpose for nanotechnology in biomedical sciences can be found in imaging, where nanodiamonds are used as (carriers for) MRI contrast agents [51,52] or as stable, fluorescent labels in lifetime imaging [36,53]. Obviously, these applications could be of great clinical importance on both diagnostics and therapeutics. Extensive pharmacokinetic analyses are required, as well as a thorough understanding of the behaviour of nanodiamonds in a physiological environment, to eventually make the translation of nanodiamond applications to the clinic. 

Here we have performed an in-depth analysis of the biological impact of fluorescent nanodiamonds uptake on HeLa cells and the generation of ROS. For future intracellular magnetic resonance measurements, this is vital background knowledge, as free radicals can influence the readout of the quantum states of the nitrogen vacancy centre. The relative safety of nanodiamonds paves the way for extensive cell experiments using FNDs and HeLa cells.

## Figures and Tables

**Table 1:** Diamond samples.

Name	NV-Centres	Average Diameter	Concentration/Condition Used	Surface Termination	Surface Potential
FND_120_	>1000 NV/particle	120 nm	1 µg/mL	Carboxylated-COOH	−20 mV
FND_70_	>300 NV/particle	70 nm	10 µg/mL, 1 µg/mL, 0.1 µg/mL, 1 µg/mL aggregated	Carboxylated-COOH	−40 mV
FND_40_	10–15 NV/particle	40 nm	1 µg/mL	Carboxylated-COOH	−45 mV
Rounded FNDs		25 nm		Carboxylated-COOH	−23 mV

**Table 2:** Primer sequences.

Target cDNA	Primer Sequence 5’->3’	Product Size, bp	Tm
Catalase	CGCAGAAAGCTGATGTCCTG	20	60.5 °C
Glutathion Reductase	TCAACGAGCTTTACCCCGAT	20	60.4 °C
SuperOxide Dismutase 1	ACAGCAGGCTGTACCAGTGC	20	59.9 °C
Caspase-3	GGGATCGTTGTAGAAGTCTAACTG	24	57.1 °C
18S	AAGGAGACTCTGGCATGCTAAC	22	58.5 °C

## Supplementary Materials

The following are available online at http://www.mdpi.com/1424-8220/18/2/355/s1, Supplementary Table S1: Antibodies for Western Blot, Supplementary Table S2: Simplified comparison of Western Blot and qPCR results, Supplementary Figure S1: Confocal images of HeLa cells used for particle and object counting (3 pages), Supplementary Figure S2: Free radical production after stimulation using LPS, Supplementary Figure S3: Viability of HeLa cells after 24 h stimulation using different concentrations of LPS, Supplementary Figure S4: Mean free radical production of nanodiamonds, Supplementary Figure S5: Morphological comparison between different concentrations of FND70 directly after uptake, 24 h after uptake and 48 h after uptake (4 pages) and Supplementary Table S3: Summary of morphological differences.
