# Supplementary material for: The Response of HeLa Cells to Fluorescent NanoDiamond Uptake
Source: Sensors (Basel). 2018 Jan 26;18(2):355. doi: 10.3390/s18020355 (PMC5855215; doi:10.3390/s18020355)
Supplement: Supplementary file 1 [file sensors-18-00355-s001.pdf]

Supplementary information

This document contains supplementary information to the research article: *The Response of HeLa cells to Fluorescent NanoDiamond uptake,* by S.R. Hemelaar et al. An outline of the supplementary tables and figures is given below:

- Supplementary Table 1: Antibodies for Western Blot
- Supplementary Table 2: Simplified comparison of Western Blot and qPCR results
- Supplementary Figure 1: Confocal images of HeLa cells used for particle and object counting (3 pages)
- Supplementary Figure 2: Free radical production after stimulation using LPS
- Supplementary Figure 3: Viability of HeLa cells after 24h stimulation using different concentrations of LPS
- Supplementary Figure 4: Mean free radical production of nanodiamonds
- Supplementary Figure 5: Morphological comparison between different concentrations of FND_{70} directly after uptake, 24 hours after uptake and 48 hours after uptake.
- Supplementary Table 3: Summary of morphological differences.

Supplementary Table 1. Primer sequences.

Antibody	Abbreviation	Dilution	Manufacturer + *reference*	Size	Blot
Primary					
αCatalase	CAT	1:1000	Abcam ab16731	60 kDa	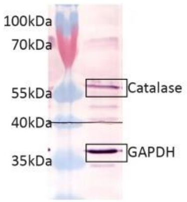
αGlutathion Reductase	GSR	1:1000	Abcam ab16801	58 kDa	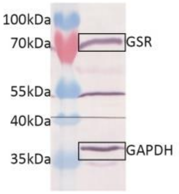
αSuperOxide Dismutase 1	SOD1	1:1000	Santa Cruz Biotechnology *sc-11407*	23 kDa	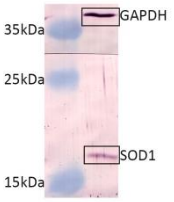
αCaspase-3	CASP	1:1000	Cell Signaling *#9662*	35 kDa	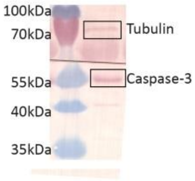
αTubulin	TUB	1:1000	Sigma-Aldrich *T4026*	55 kDa	
αGlyceraldehyde-3-Phosphate DeHydrogenase	GAPDH	1:1000	Abcam *ab9484*	36 kDa	
Secondary					
Mouse αRabbit	MαRb	1:1000	Jackson ImmunoResearch 211-005-109		
Goat αMouse	GαM	1:1000	Jackson ImmunoResearch *115-005-071*		
Tertiary					
Goat αMouse-AP	GαM-AP	1:1000	Bio-Rad *#1706520*		
Rabbit αGoat-AP	RbαG-AP	1:1000	Jackson ImmunoResearch 305-055-046		

Supplementary Table 2. Simplified comparison of Western Blot and qPCR results.

Sample	Catalse		GSR		SOD1		Caspase 3	
	qPCR	Western Blot	qPCR	Western Blot	qPCR	Western Blot	qPCR	Western Blot
10 ug FND 70	≈	↑	↓	↓	↓	↓	↓	↑↑
10 µg FND 70 T=24	≈	≈	≈	↑	↓	↑↑	≈	↓
1 µg FND 70	↓	↑	↓	↓	↓	≈	↓	↑
1 µg FND 70 T=24	≈	↓	≈	≈	≈	↑	≈	↓
0.1 µg FND 70	↓	↑	↓	↓	↓↓	≈	↓	≈
0.1 µg FND 70 T=24	↓	↓	↓	≈	↓	↑	≈	↓
1mM H2O2	≈	≈	↓	↓	↓	≈	↓	↑
1mM H2O2 T=24	↓	↑↑	↓	↑↑	↓	↑	≈	↓↓
200µM H2O2	≈	≈	↓	↓	↓	≈	↑	≈
200µM H2O2 T=24	≈	↓	≈	≈	≈	≈	≈	↓
40µM H2O2	≈	≈	≈	↓	↓	≈	≈	≈
40µM H2O2 T=24	≈	↓	≈	↑	≈	≈	↑	↓
1 µg FND120	≈	↑↑	≈	≈	↓	≈	↓	≈
1 µg FND40	≈	≈	↑	≈	↑	≈	↑	↑
1 µg FND 70 aggregated	≈	≈	≈	≈	↑	↑	≈	↑
1 µg rounded FNDs	≈	≈	≈	≈	≈	↑↑	≈	↑↑

↑↑ reflects a higher than 0.75% increase, ↑ reflects a 0.25 to 0.75 % increase, ≈ reflects a similar comparison 0.25% to -0.25%, ↓ reflects a -0.25% to -0.75% decrease, ↓↓ reflects a bigger than -0.75% decrease.

0.1 µg FND 70nm

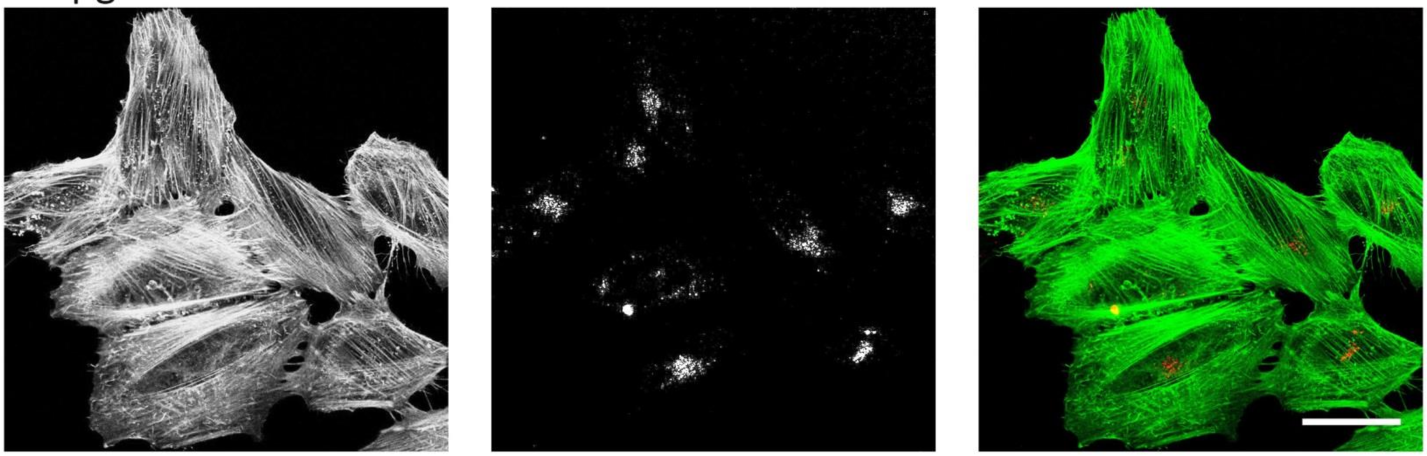

0.1 µg FND 70nm T=24

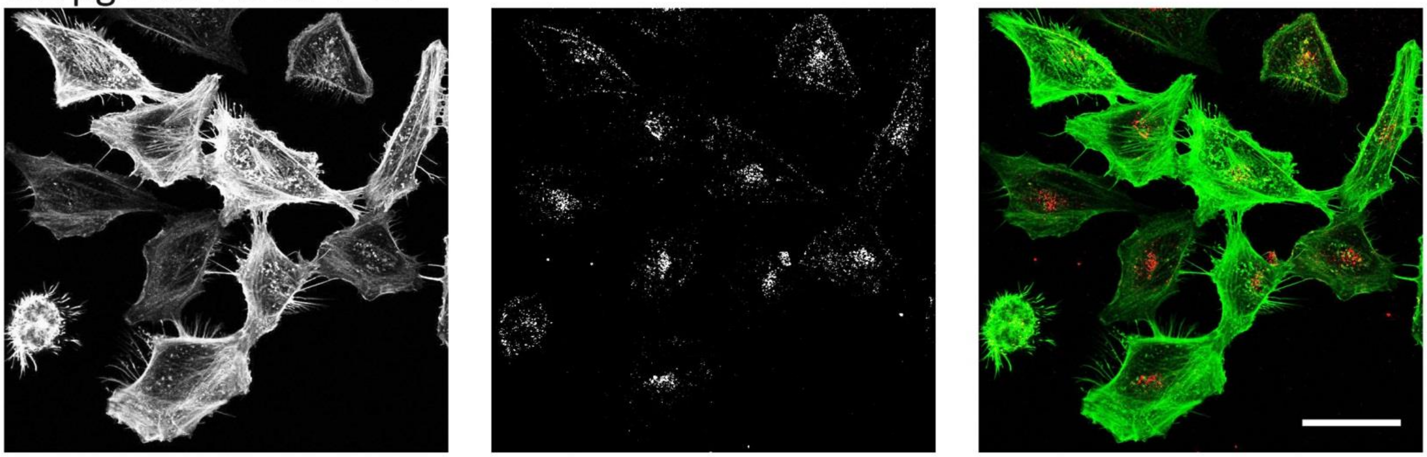

1 µg FND 70nm

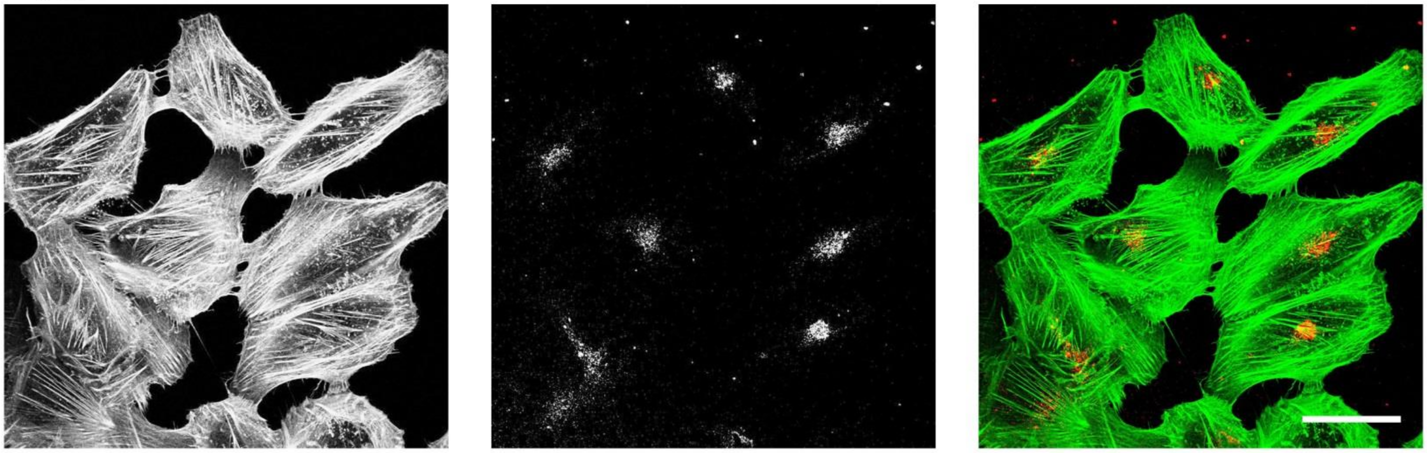

1 µg FND 70nm T=24

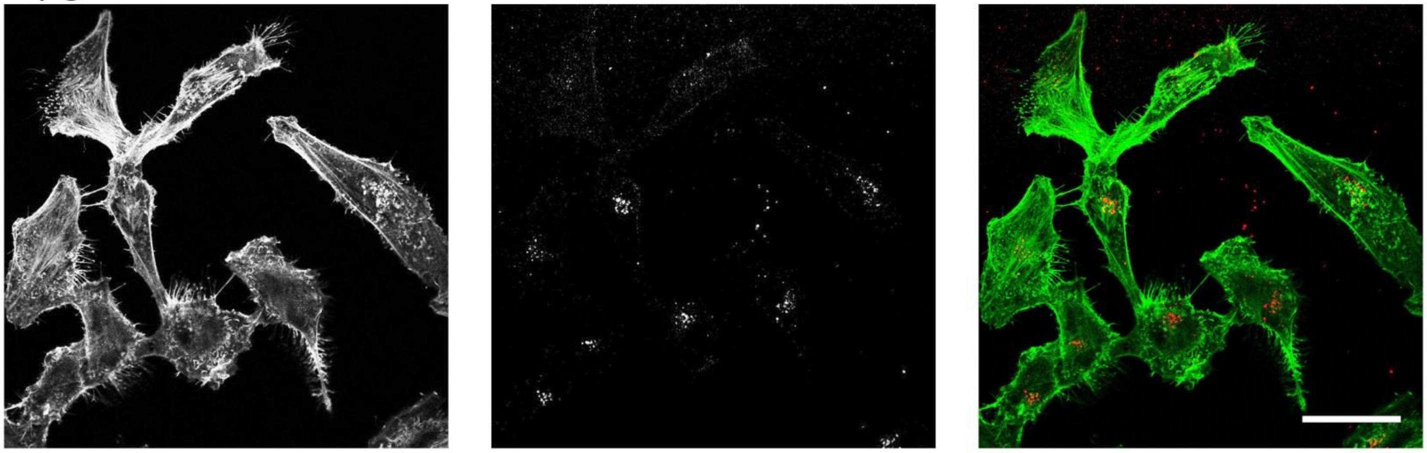

10 μg FND 70nm

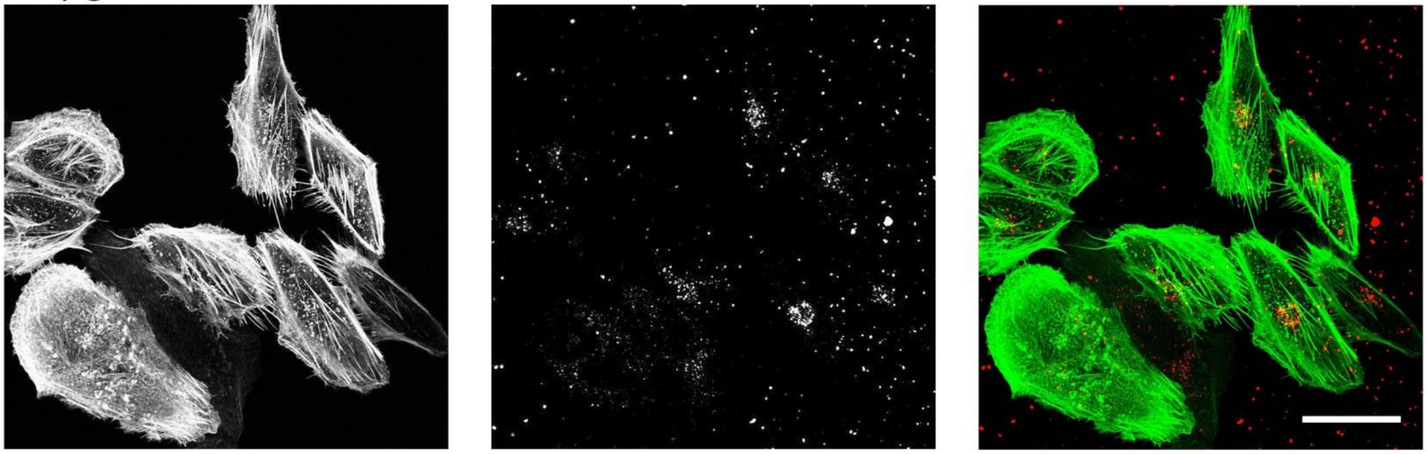

10 μg FND 70nm T=24

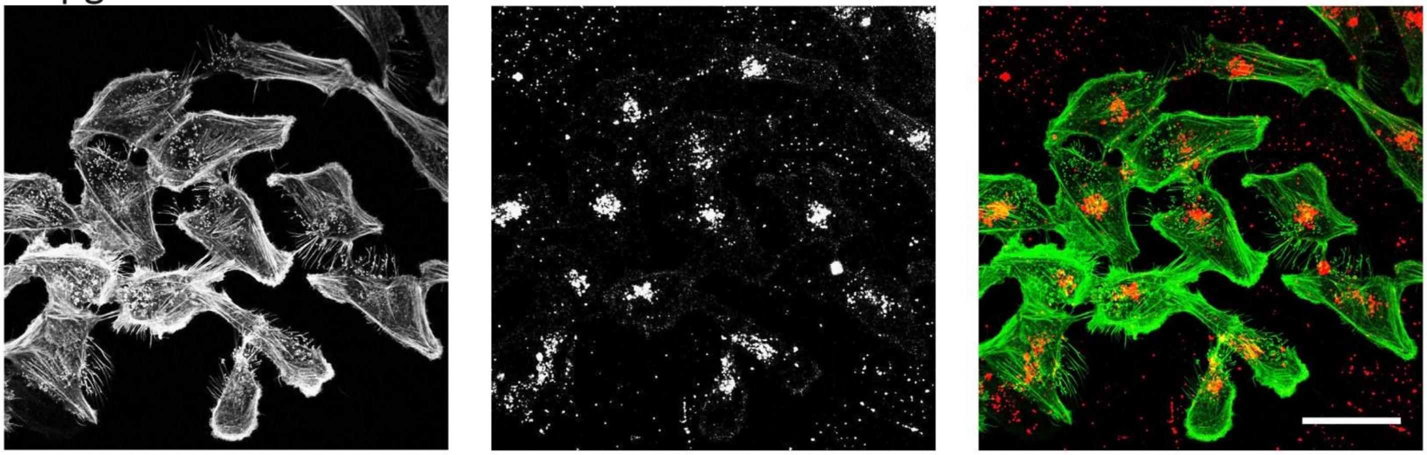

1 μg aggregated FND 70nm

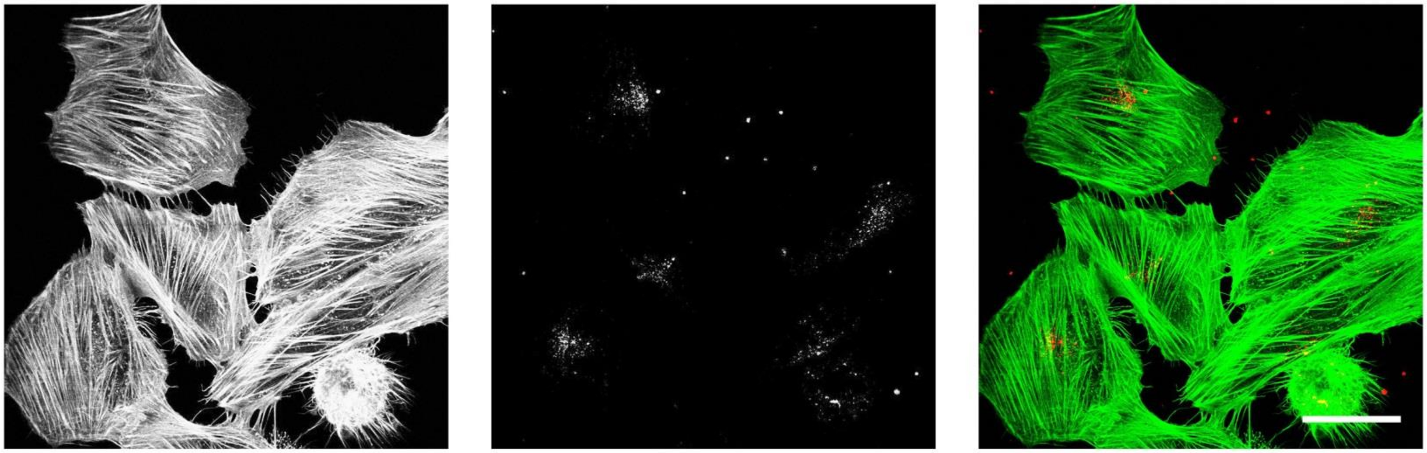

1 μg rounded FNDs

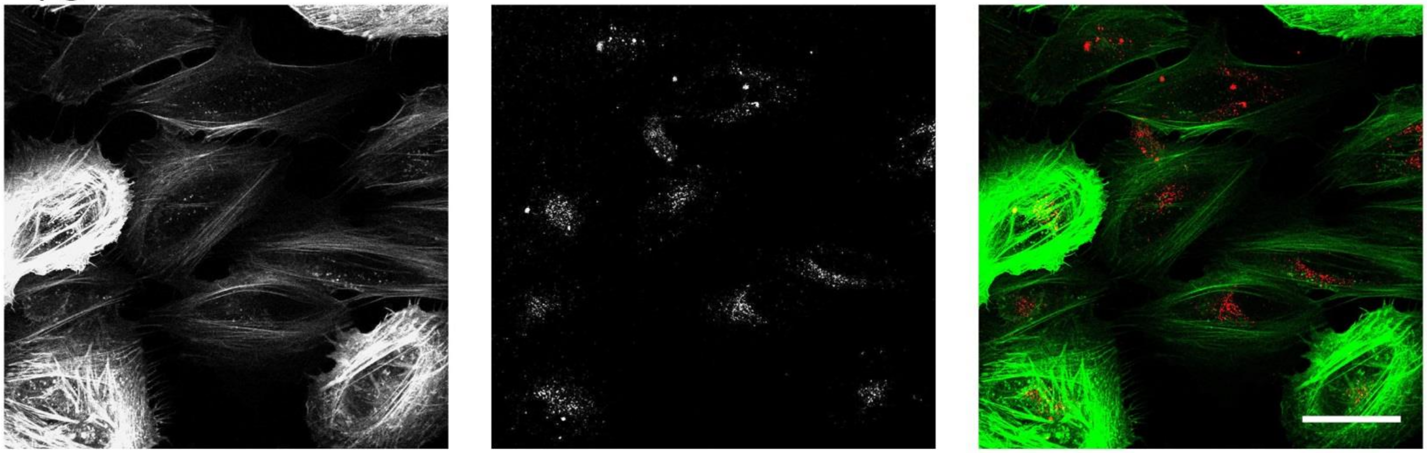

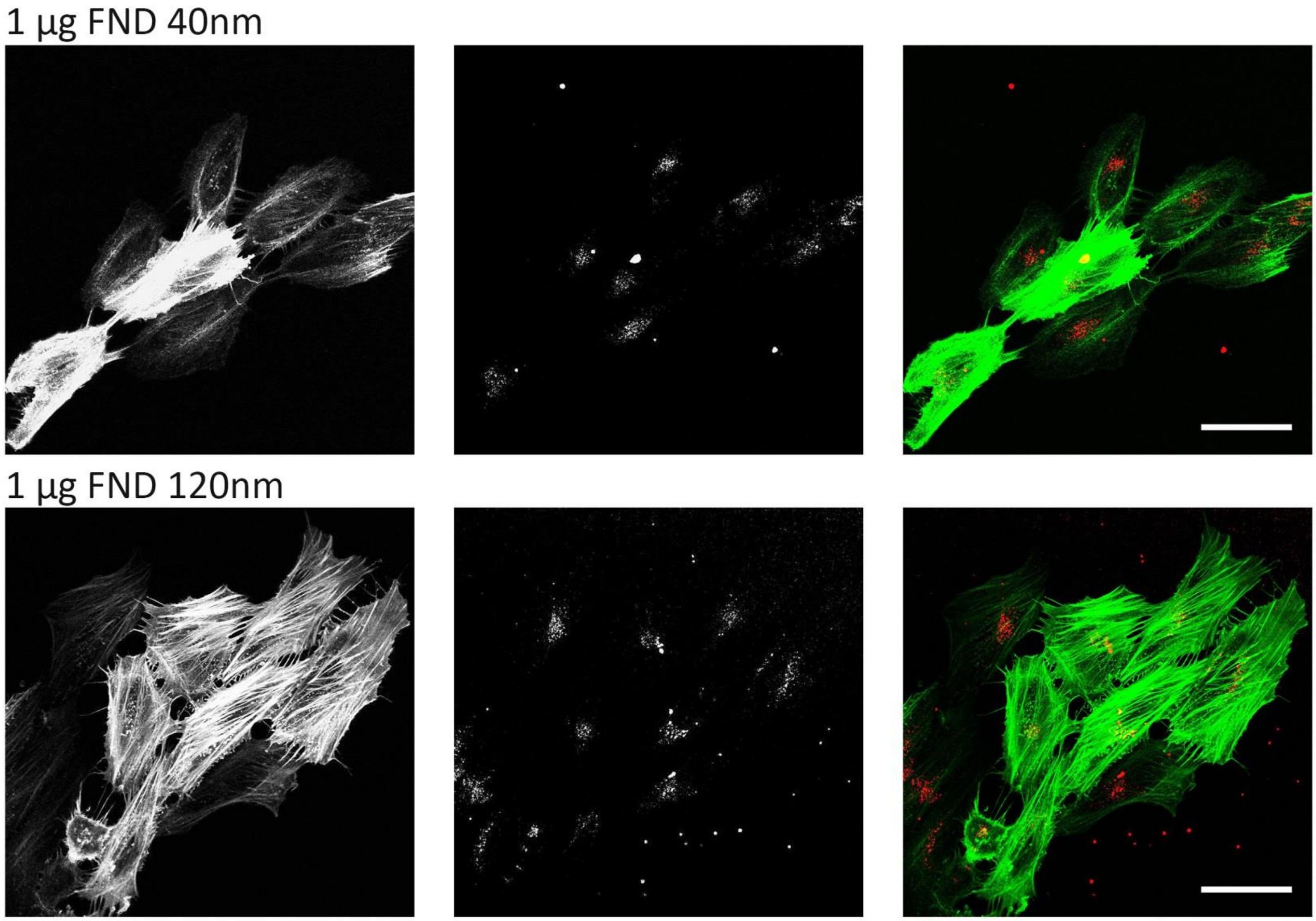

Supplementary Figure 1. Confocal images of HeLa cells used for particle and object counting. Each horizontal line of panels represents one condition, the condition is listed on top of the left image. All images are cross sections at the base of the HeLa cells. Left images are a greyscale of the signal coming from FITC-Phalloidin, which labels the actin cytoskeleton. The center images present the signal of FNDs, also in grey scale. In the right images, an overlay is made from both the FITC signal (in green) and the FND signal in red. The scale bar represents 50µm.

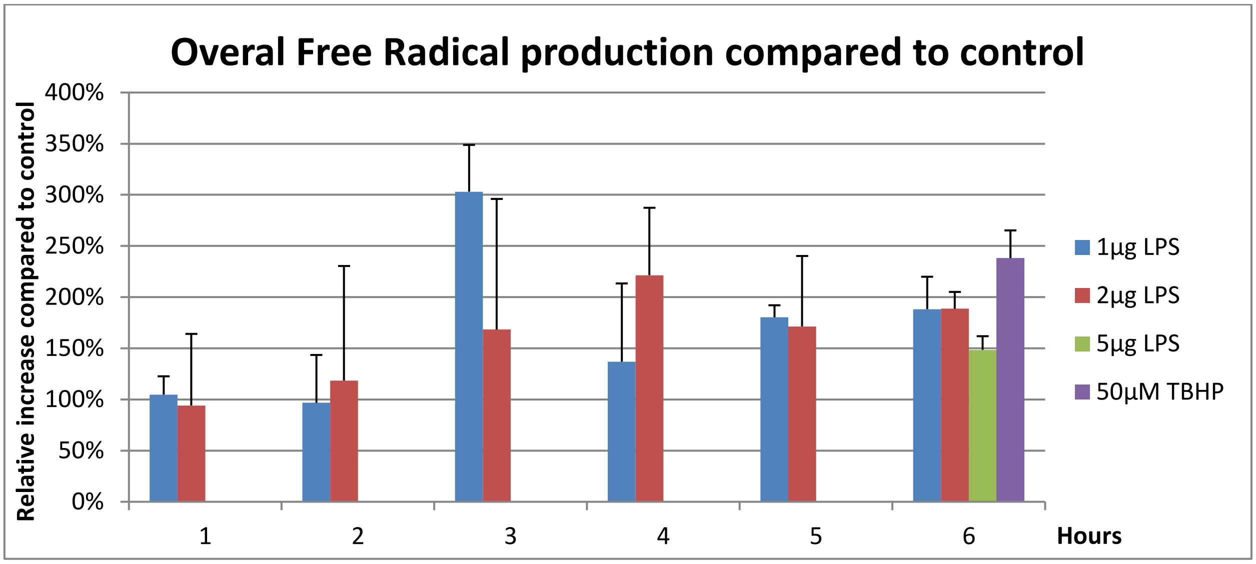

Supplementary Figure 2. Free radical production after stimulation using LPS. In order to test how big the increase of free radical production after stimulation with LPS was, we incubated HeLa cells with DCFDA and stimulated them with different concentrations of LPS for different amounts of time. Then we measured the generated fluorescent signal with an FLUOstar Omega Microplate Reader and related the signal to a non-stimulated control. This gave values of fold increase, which are expressed in percentages. As a positive control we used 50 µM TBHP, provided by the manufacturer of the DCFDA kit. Although the free radical production was increased after LPS stimulation, this difference was not nearly as big as when we stimulated the cells using hydrogen peroxide. Because of this the genetic and protein differences will be smaller to see and more difficult to relate. Therefore we chose to stick with H_2O_2 as a stimulant. Error bars show the standard deviation.

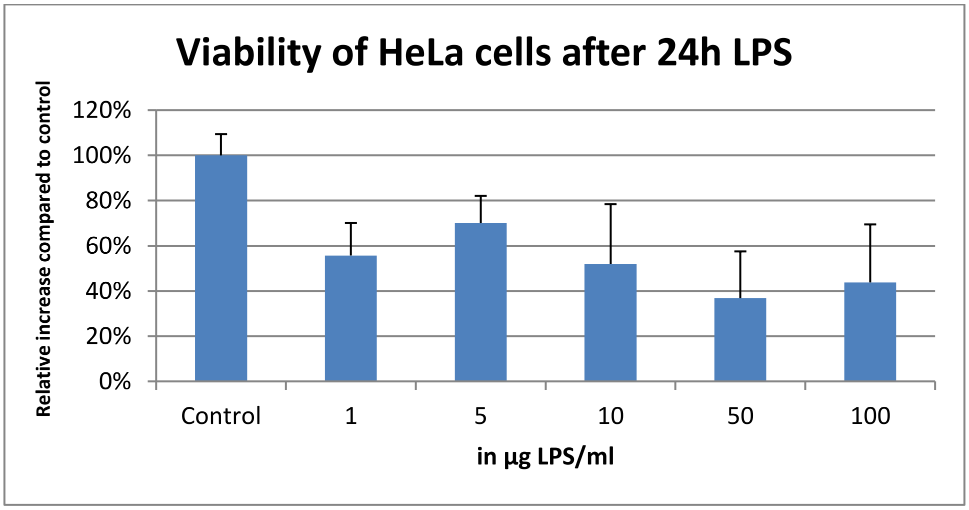

Supplementary Figure 3. Viability of HeLa cells after 24h stimulation using different concentrations of LPS. We stimulated HeLa cells for 24 hours using LPS to see what was the effect on the viability of HeLa cells. Even at low concentrations of 1µg LPS / ml the cellular viability was reduced by half. This rendered a time as long as 24 hours not viable for LPS experiments. Error bars show the standard deviation.

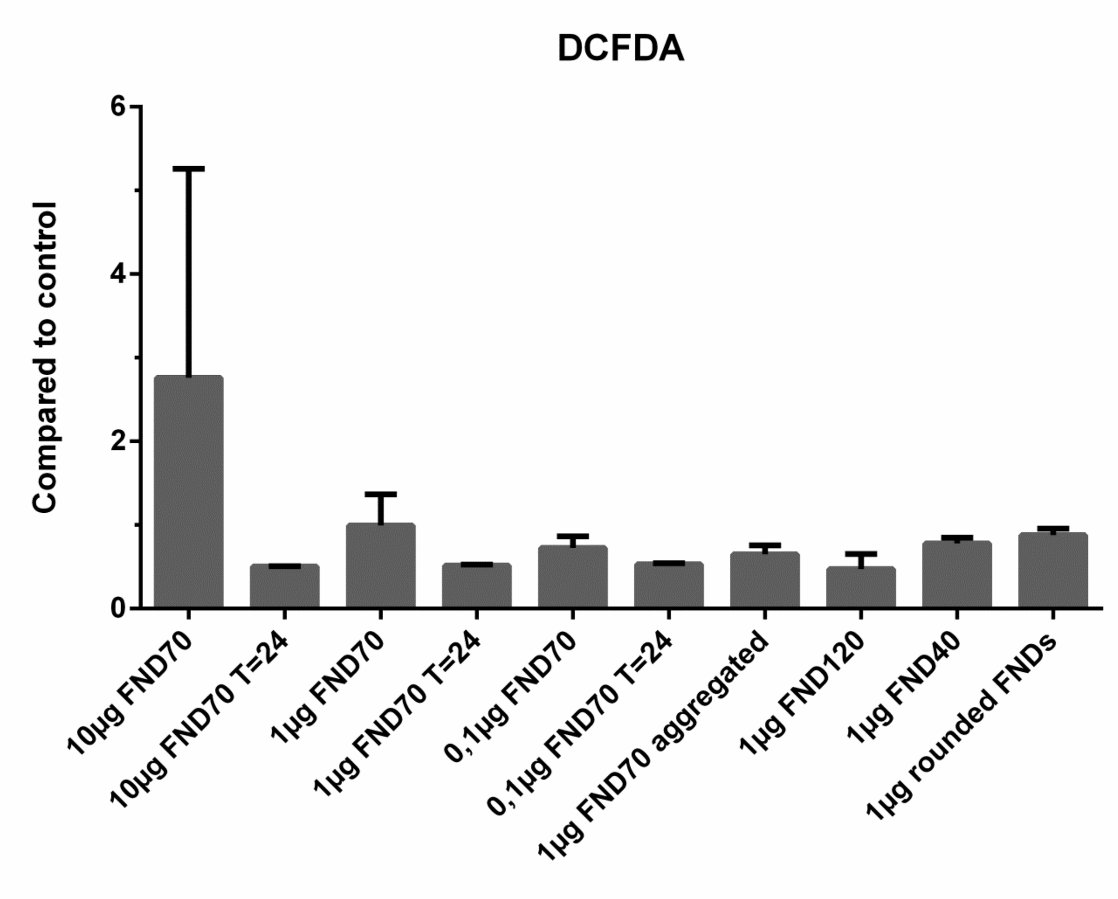

Supplementary Figure 4. Mean free radical production of nanodiamonds. A close up of Figure 4 shows a more detailed overview of the (non-significant) changes to total free radical production in HeLa cells. Error bars show the standard deviation.

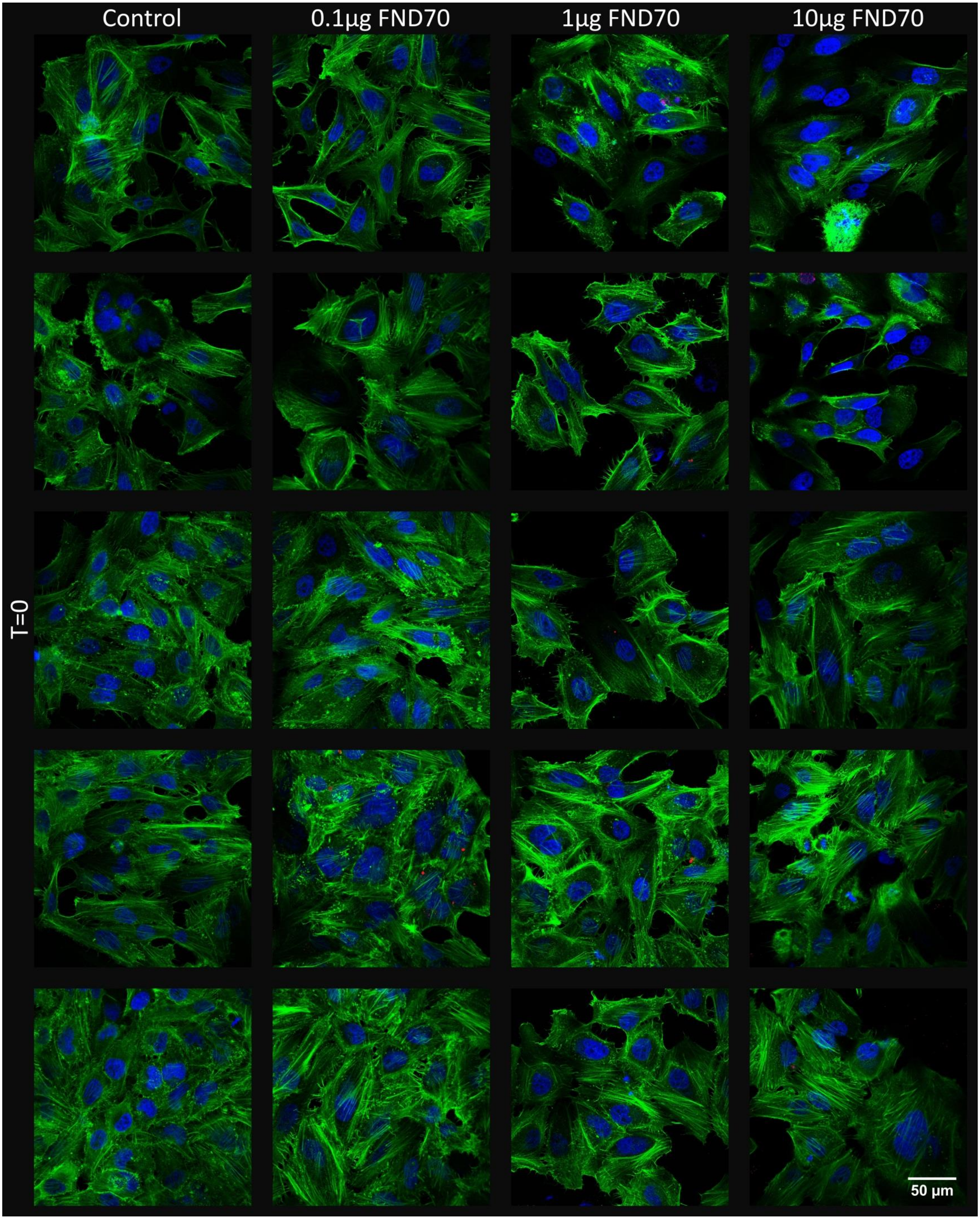

Control
0.1μg FND70
1μg FND70
10μg FND70
T=0
50 μm

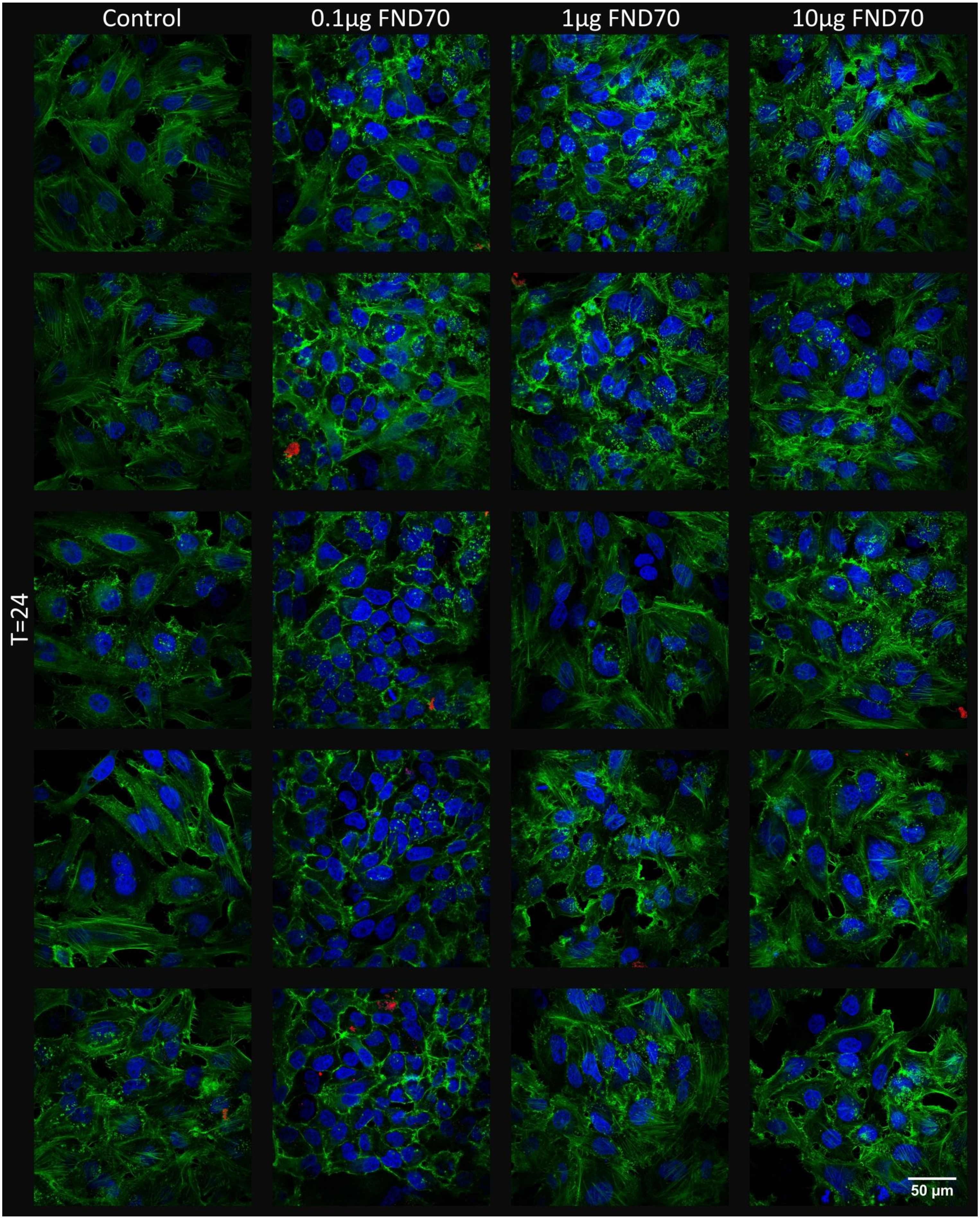
Control
0.1μg FND70
1μg FND70
10μg FND70
T=24
50 μm

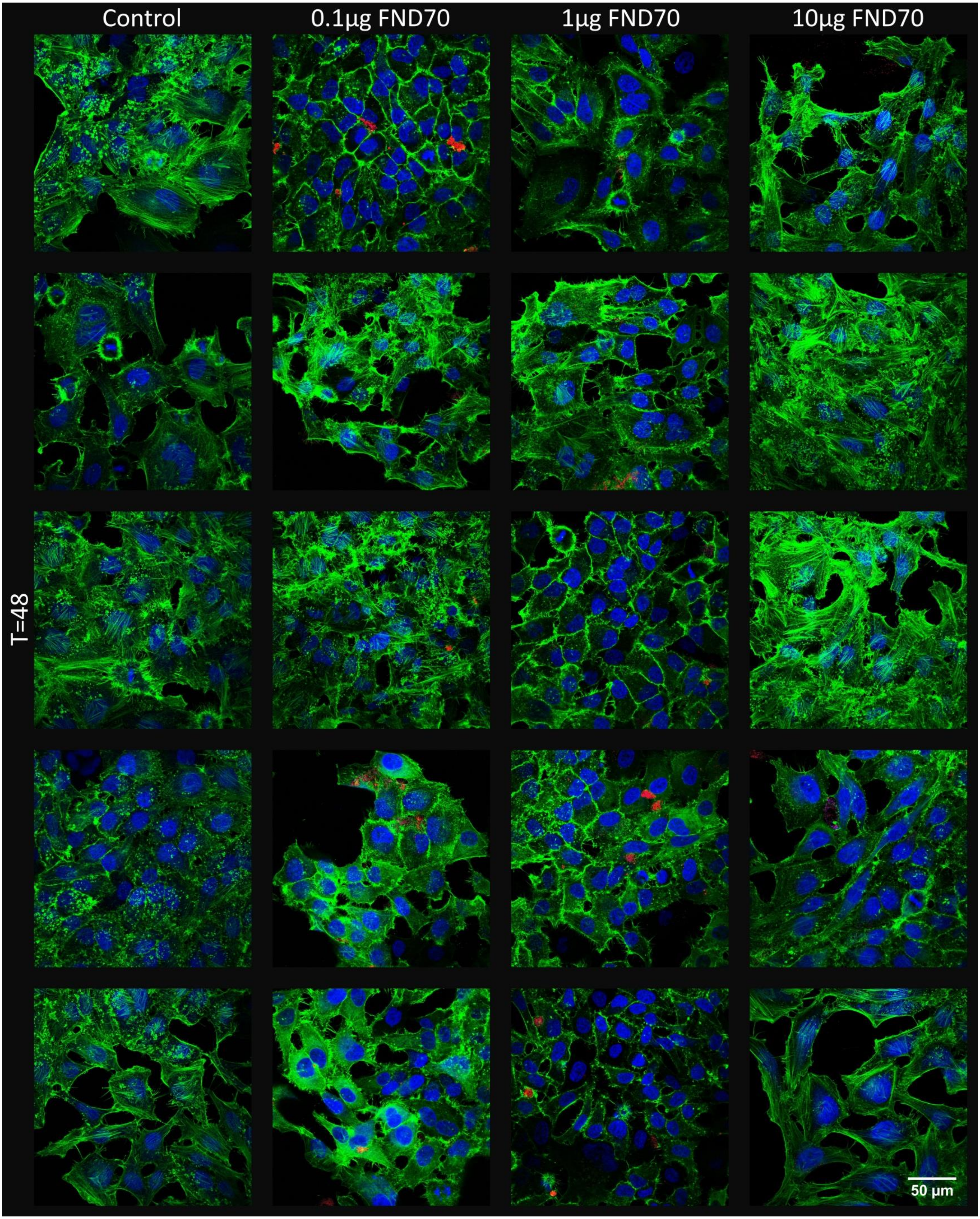
Control
0.1µg FND70
1µg FND70
10µg FND70
T=48
50 µm

Supplementary Figure 5. Morphological comparison between different concentrations of FND_{70} directly after uptake, 24 hours after uptake and 48 hours after uptake. After incubation of HeLa cells with different concentrations of FND_{70}, we either immediately fixed the cells in 3.7% PFA or we incubated them for 24 or 48 hours more with supplemented DMEM medium and then fixed them. The resulting samples were stained with phalloidin-FITC and DAPI and imaged using a ZEIS LSM780 microscope. Of each sample 5 pictures were made to obtain enough cells to count. If available, samples from Supplementary Figure 1 were also taken into account. The scale bar represents 50µm and is the same for all images. The results are summarized in Supplementary Table 3 and can be seen as an estimation of the effect of nanodiamonds on the cytoskeletal morphology of the cells. The term 'other' refers to cells which don't look normal but are not specified further. Sometimes this might also refer to cells in mitosis. The other morphological changes are indicators of an early onset of apoptosis (Taylor, R. C.; Cullen, S. P.; Martin, S. J. Apoptosis: Controlled demolition at the cellular level. *Nat. Rev. Mol. Cell Biol.* 2008, *9*, 231–241.). Although some samples show early signs of apoptosis, this can also be found in the control sample, therefore we attribute this to a natural variation.

Supplementary Table 3. Summary of morphological differences.

	Protrusions	Uneven filament distribution	Rounding of cells	Blebbing	Other
Control T=0	1%	0%	2%	0%	7%
Control T=24	2%	7%	2%	0%	1%
Control T=48	2%	1%	3%	0%	1%
0.1µg FND70 T=0	0%	0%	0%	0%	5%
0.1µg FND70 T=24	0%	0%	0%	0%	2%
0.1µg FND70 T=48	2%	0%	1%	0%	3%
1µg FND70 T=0	0%	0%	0%	0%	3%
1µg FND70 T=24	1%	1%	1%	0%	2%
1µg FND70 T=48	0%	2%	2%	0%	1%
10µg FND70 T=0	0%	4%	4%	0%	9%
10µg FND70 T=24	0%	0%	0%	0%	1%
10µg FND70 T=48	2%	2%	0%	0%	0%
